# From Enforcement to Resilience: Evaluating Indonesia's One Roof Enforcement System to Combat Illegal Fishing in the North Natuna Sea

**DOI:** 10.12688/f1000research.179511.1

**Published:** 2026-05-12

**Authors:** Handrisal Handrisal, Armaidy Armawi, Heribertus Jaka Triyana, I Made Andi Arsana

**Affiliations:** 1 Doctoral Program in National Resilience Science, Gadjah Mada University Graduate School, Yogyakarta, Special Region of Yogyakarta, Indonesia; 2 Department of Government Science, Universitas Maritim Raja Ali Haji, Riau Islands, Riau, Indonesia; 3 Faculty of Philosophy, Gadjah Mada University, Yogyakarta, Special Region of Yogyakarta, Indonesia; 4 Faculty of Law, Gadjah Mada University, Yogyakarta City, Special Region of Yogyakarta, Indonesia; 5 Gadjah Mada University Faculty of Engineering, Yogyakarta, Special Region of Yogyakarta, Indonesia

**Keywords:** IUU Fishing; Maritime Governance; Multi-Agency Enforcement; OECD Policy Evaluation; Maritime Resilience; North Natuna Sea; Indonesia

## Abstract

**Background:**

Illegal, unreported, and unregulated (IUU) fishing in the North Natuna Sea represents a complex governance challenge situated at the intersection of geopolitical contestation, transnational economic dynamics, and institutional fragmentation. As a strategically contested maritime space, the region exposes limitations in conventional enforcement-based approaches. Indonesia has responded through the establishment of Task Force 115 under a one roof enforcement system, integrating multiple maritime agencies to strengthen law enforcement. However, the extent to which this approach contributes to long-term maritime resilience remains underexplored.

**Methods:**

This study adopts a qualitative case study design to evaluate Indonesia’s multi-agency enforcement model. Data were collected through 15 semi-structured interviews with key informants from maritime enforcement institutions, document analysis of regulatory and operational frameworks, and limited participatory observation. Data were analyzed using inductive thematic analysis guided by the OECD policy evaluation framework, encompassing relevance, coherence, effectiveness, efficiency, impact, and sustainability, and interpreted through a maritime resilience perspective.

**Results:**

The findings show that the one roof enforcement system enhances horizontal coordination, reduces institutional overlap, and strengthens short-term deterrence capacity. This is evidenced by increased vessel apprehensions and a temporary decline in foreign fishing activities during periods of intensified patrol operations. However, policy performance remains highly dependent on resource inputs, including budget allocation, technological support, and political prioritization. While Indonesia demonstrates strong absorptive capacity in responding to maritime violations, adaptive capacity remains limited due to insufficient institutional learning and technological integration, and transformative capacity is constrained by weak incorporation of community-based fisheries governance and broader structural reforms.

**Conclusions:**

Indonesia’s multi-agency enforcement model constitutes a necessary but insufficient foundation for achieving sustainable maritime resilience. Long-term effectiveness requires a transition from enforcement-centric governance toward adaptive, integrated, and resilience-based policy approaches. Strengthening institutional continuity, technological modernization, cross-sectoral integration, and community engagement is essential to address IUU fishing in contested maritime environments.

## 1. Introduction

Illegal, unreported, and unregulated (IUU) fishing has long been recognized as one of the most serious challenges in global ocean governance due to its far-reaching impacts on the sustainability of fish stocks, the economic stability of coastal communities, and the maritime security of coastal states. In the context of a large archipelagic state such as Indonesia, this threat extends far beyond the fisheries sector, intersecting directly with issues of sovereignty, national resilience, and regional geopolitics. The North Natuna Sea represents a particularly salient example of this complexity. The area is not only endowed with strategically significant fisheries resources but is also situated within a sensitive geopolitical landscape where competing claims, economic interests, and transboundary activities converge. As such, the North Natuna Sea constitutes aß contested maritime space vulnerable to IUU fishing practices, while simultaneously serving as a testing ground for state capacity in law enforcement and sustainable maritime resilience-building. ^1, 2^

The Government of Indonesia has undertaken various measures to address the IUU fishing threat, particularly through the strengthening of maritime law enforcement and the establishment of cross-agency coordination mechanisms. The legal foundation of fisheries governance is primarily anchored in *Undang-Undang Nomor 45 Tahun 2009 tentang Perikanan,* ^3^ which provides the overarching regulatory framework for fisheries management and enforcement. Surveillance and monitoring mechanisms are further regulated under *Peraturan Pemerintah Nomor 60 Tahun 2007 tentang Pengawasan Sumber Daya Perikanan dan Kelautan.* ^4^

A pivotal institutional initiative was the creation of Task Force 115 ( *Satgas 115*) through *Peraturan Presiden Nomor 115 Tahun 2015 tentang Satuan Tugas Pemberantasan Penangkapan Ikan Secara Ilegal.* ^5^ This regulation institutionalized an extraordinary, integrated enforcement mechanism combining the authority and resources of key institutions, including the Ministry of Marine Affairs and Fisheries, the Indonesian Navy, the Maritime Security Agency ( *Badan Keamanan Laut*), established under *Peraturan Presiden Nomor 178 Tahun 2014,* ^6^ the National Police, and the Attorney General’s Office. The organizational structure and operational procedures of *Satgas 115* were subsequently refined under *Peraturan Menteri Kelautan dan Perikanan Nomor 24/PERMEN-KP/2020.* ^7^

This institutional arrangement reflects a shift from sectoral approaches toward a multi-agency governance model under a “one roof enforcement system.” Empirically, the approach has been associated with intensified enforcement actions, including high-profile vessel seizures and scuttling operations, ^8^ and a temporary reduction in the presence of foreign fishing vessels. ^9, 10^ However, the continuity and effectiveness of *Satgas 115* have been influenced by political and administrative dynamics, including periods of institutional stagnation and reactivation. ^11^ These developments suggest that operational success remains highly contingent upon patrol intensity, budgetary support, and political stability, revealing a potential gap between short-term enforcement outputs and the long-term objective of adaptive and sustainable maritime resilience.

From an academic standpoint, studies on IUU fishing in Indonesia have largely been dominated by law enforcement and normative compliance perspectives, focusing on legal instruments, sanctions, and institutional capacity. ^9^ While such analyses are essential, they tend to frame IUU fishing primarily as a law enforcement problem, without sufficiently situating it within broader regional political economy dynamics, institutional fragmentation, and cross-sectoral governance challenges. Contemporary international scholarship increasingly conceptualizes maritime crime including fisheries offenses as embedded within complex global systems of demand, ecological degradation, and uneven regulatory capacity. ^12– 14^ In Indonesia’s case, economic losses in the Natuna Exclusive Economic Zone have been shown to be substantial, ^15^ further underscoring the structural dimensions of the problem.

Moreover, maritime security studies have called for moving “beyond seablindness” by integrating governance, security, and socio-ecological perspectives. ^1^ This is particularly relevant in the North Natuna Sea, where maritime enforcement intersects with Indonesia’s broader maritime strategy ^16^ and evolving defense posture, ^17^ as well as with shifting geopolitical configurations in the Indo-Pacific. ^18, 19^ At the local level, resilience and preparedness among coastal communities in Natuna also shape the sustainability of maritime governance outcomes. ^20^ Despite these developments, there remains limited scholarship that systematically evaluates Indonesia’s IUU fishing policy by integrating multi-agency governance, one roof enforcement mechanisms, public policy evaluation frameworks, and maritime resilience concepts particularly in a strategically sensitive maritime zone such as the North Natuna Sea.

This study addresses this gap by providing a comprehensive evaluation of Indonesia’s multi-agency approach to combating IUU fishing in the North Natuna Sea. Unlike studies that assess policy success solely based on the number of enforcement actions or vessels apprehended, this article situates *Satgas 115* within the OECD policy evaluation framework, emphasizing relevance, coherence, effectiveness, efficiency, impact, and sustainability. This evaluative lens is combined with the concept of maritime resilience, understood as the capacity of maritime systems to absorb pressures, adapt to change, and transform governance structures sustainably. Such an approach enables a more nuanced analysis of whether enforcement policies merely respond to short-term threats or contribute to strengthening the structural capacity of Indonesia’s maritime governance system.

Accordingly, this research seeks to answer three principal questions. First, what are the characteristics and dynamics of IUU fishing threats in the North Natuna Sea when understood as a multidimensional phenomenon shaped by structural, political-economic, institutional, and geopolitical factors? Second, to what extent is the multi-agency approach embodied in *Satgas 115* relevant, coherent, and effective in addressing these threats, and what limitations emerge in its implementation? Third, what are the broader implications of this policy for the development of Indonesia’s maritime resilience, particularly in the transition from an enforcement-centric paradigm toward a resilience-based approach?

The primary scholarly contribution of this article lies in integrating public policy evaluation and maritime resilience frameworks in analyzing Indonesia’s response to IUU fishing. By employing the OECD evaluation criteria, this study moves beyond assessing enforcement outputs and instead reveals the structural and institutional constraints shaping policy sustainability. Furthermore, by conceptualizing the North Natuna Sea as a contested maritime space embedded within complex geopolitical and socio-ecological systems, ^15^ this article enriches international debates on how large archipelagic states address fisheries crime under conditions of strategic competition. Practically, the findings are expected to inform the formulation of more adaptive, integrated, and resilience-oriented maritime policies in Indonesia, while contributing conceptually to the broader development of maritime security and governance studies.

## 2. Methods

### 2.1. Research design

This study adopts a qualitative research approach employing a case study strategy. Qualitative research is particularly suitable for exploring contextualized social phenomena through non-numeric data such as narratives, documents, and observed interactions, and is especially effective in addressing complex “how” and “why” research questions situated within real-life settings. ^21, 22^

A case study strategy was selected because it enables an in-depth examination of multi-agency coordination dynamics in combating IUU fishing within the North Natuna Sea an empirically bounded, contemporary, and geopolitically sensitive maritime space. Given the intersection of sovereignty, security, and resource governance in this region, a case study approach allows for the integration of multiple sources of evidence and institutional perspectives. ^23, 24^ The North Natuna Sea constitutes a complex maritime arena shaped by geopolitical contestation in the South China Sea, ^25, 26^ and long-standing maritime community interactions in Southeast Asia, ^27^ making it analytically suitable for qualitative case-based inquiry.

### 2.2. Data sources

This study utilizes both primary and secondary data.

Primary data were obtained through in-depth interviews with key informants, including officials from maritime law enforcement agencies, fisheries supervisors, and local actors involved in the implementation of IUU fishing countermeasures. These interviews were designed to capture institutional experiences, operational challenges, and perceptions of multi-agency coordination within the broader maritime security framework. ^24, 28^

Secondary data include national and regional policy documents, official institutional reports, inter-agency coordination records, and recent scholarly literature addressing maritime resilience, governance, and fisheries enforcement. Literature examining geopolitical dynamics, ^25^ maritime administrative control, ^26^ border area development strategies, ^28^ and ecological-economic fisheries profiles, ^29– 31^ were incorporated to contextualize enforcement within broader structural and socio-ecological dynamics. Additionally, research on resource deployment and maritime operational optimization ^32^ and global fisheries externalities ^33^ informed the analytical framing of enforcement effectiveness and structural pressures. The combination of primary and secondary sources ensures methodological triangulation, thereby strengthening interpretive validity and analytical depth. ^21, 22^

### 2.3. Data collection techniques

Data collection techniques included semi-structured interviews, document analysis, and limited participatory observation of inter-agency coordination processes. Semi-structured interviews were designed to elicit in-depth perspectives on coordination mechanisms, institutional constraints, enforcement strategies, and the perceived contribution of multi-agency approaches to maritime resilience. A total of 15 key informants were interviewed, representing fisheries supervision authorities (PSDKP), the Indonesian Navy, *Badan Keamanan Laut*, the Maritime Police, prosecutorial institutions, regional government officials, and civil society monitoring organizations. Informants were selected using purposive sampling based on their direct institutional involvement in IUU enforcement and maritime security coordination in the North Natuna Sea. Interviews continued until thematic saturation was achieved. Document analysis involved systematic review of strategic plans, maritime patrol standard operating procedures, enforcement reports, and institutional mandates to understand the regulatory and operational context of policy implementation.

Participatory observation allowed the researcher to document patterns of interaction, decision-making processes, and coordination dynamics among maritime enforcement institutions. Such multi-method qualitative techniques are consistent with established qualitative design principles that emphasize contextual immersion and data richness. ^21^

### 2.4. Literature search and inclusion criteria

The literature review and secondary data search were conducted using systematic inclusion and exclusion criteria. Inclusion criteria comprised:

1. Publications between 2018 and 2025;
2. Open-access availability;
3. Direct relevance to IUU fishing, maritime governance, geopolitics of the South China Sea, border security, or multi-agency coordination;
4. Publications in English or Indonesian.

Studies published before 2018, lacking relevance to institutional coordination or IUU fishing, or unavailable for public access were excluded. The screening process followed critical appraisal principles for qualitative research to ensure credibility and relevance. ^22^

### 2.5. Unit of analysis and sampling

The unit of analysis includes inter-agency interactions, institutional policy documentation, and narratives from key informants involved in the implementation of IUU fishing countermeasures. Institutions examined include maritime enforcement bodies and coordinating agencies operating within Indonesia’s maritime governance architecture. Purposive sampling was employed to select key informants based on their direct involvement in enforcement coordination, strategic decision-making, or operational implementation. This sampling strategy ensures depth of experiential insight and relevance to the research questions. ^21^

**Ethical Approval**

This study has received ethical approval from the Research Division, Graduate School, Universitas Gadjah Mada, under approval number 8322/UN1/SPs.1.1/AKM/PT/12/2025. The research involved qualitative data collection through semi-structured interviews with professional informants from relevant maritime and enforcement institutions. Prior to participation, all informants were provided with detailed information regarding the purpose, procedures, and voluntary nature of the study. Oral informed consent was obtained from all participants before data collection, as described in the Methods section (Data Collection Techniques). The use of oral consent was considered appropriate due to the professional status of the informants and the institutional context of the interviews, where formal written consent could potentially reduce openness, create administrative barriers, or raise sensitivity concerns related to organizational protocols and confidentiality. The consent process was documented by the researcher at the beginning of each interview. No participants in this study were minors; therefore, parental consent or assent procedures were not required. To ensure confidentiality and privacy, all participants’ identities were anonymized during transcription, analysis, and reporting. All procedures were conducted in accordance with applicable ethical standards for research involving human participants.

### 2.6. Data analysis

Data were analyzed using inductive thematic analysis complemented by narrative synthesis. Thematic analysis involved systematic identification, coding, and categorization of recurring patterns across interview transcripts, policy documents, and observation notes. The analytical process included data familiarization, initial coding, theme development, thematic refinement, and interpretative synthesis within the conceptual framework of multi-agency governance and maritime resilience. Narrative synthesis was employed to integrate thematic findings into a coherent explanatory account linking empirical evidence with policy and geopolitical contexts. This approach enables interpretation of how institutional coordination functions in practice and how it contributes to broader maritime resilience objectives.

## 3. Results

The findings are structured based on inductive thematic analysis of interview transcripts, policy documents, and observational notes. Four major themes emerged consistently across data sources: (1) the multidimensional and geopolitical character of IUU fishing in the North Natuna Sea; (2) the operational effectiveness and institutional limits of the one roof enforcement system; (3) the structural gap between enforcement success and long-term maritime resilience; and (4) pathways toward adaptive and transformative maritime governance.

**Figure 1.**
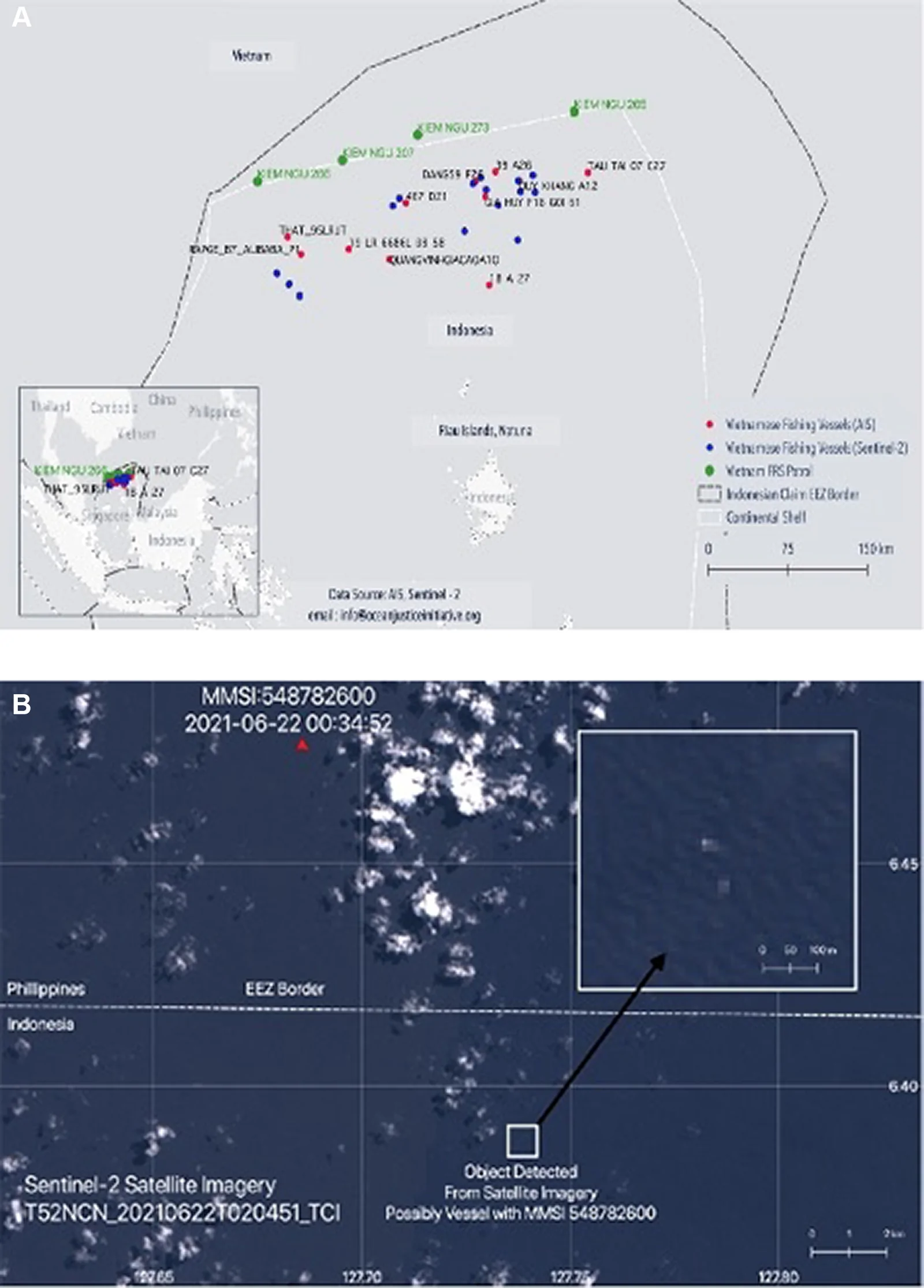
Empirical detection and enforcement mapping in the north natuna sea. *a.* Spatial Distribution of Suspected Vietnamese IUU Fishing Vessels within Indonesia’s EEZ. b. Satellite-Based Vessel Identification and MMSI Confirmation near the EEZ Boundary. **Source:** Indonesia Ocean Justice Initiative (IOJI), *Ancaman IUU Fishing dan Keamanan Laut Indonesia*, August 2021.

Figure 1 provides geospatial evidence of suspected Vietnamese IUU fishing activities within Indonesia’s claimed Exclusive Economic Zone (EEZ) in the North Natuna Sea. Panel (a) illustrates the spatial clustering of detected vessels based on satellite imagery and AIS data, highlighting their proximity to Indonesia’s EEZ boundary and areas of intensified patrol operations. Panel (b) presents high-resolution Sentinel-2 satellite imagery indicating object detection and MMSI-linked vessel identification, demonstrating how remote sensing technologies support verification and enforcement processes.

### 3.1. IUU fishing as a transnational and geopolitical phenomenon

The findings indicate that IUU fishing in the North Natuna Sea cannot be understood solely as a fisheries law violation. Interview data reveal that illegal fishing activities are closely intertwined with broader geopolitical contestation in the South China Sea. Informants from maritime enforcement agencies emphasized that incursions by foreign fishing vessels frequently coincide with heightened diplomatic tensions and administrative assertions by external actors. This aligns with scholarship highlighting the strategic intersection of geopolitics, sustainability, and maritime leadership in the South China Sea. ^25, 34^ The administrative consolidation strategies observed in the region further demonstrate how fisheries activities can function as instruments of state presence and maritime influence.

Moreover, respondents identified patterns consistent with transnational organized fisheries crime. These findings reflect broader analyses showing that illegal fishing networks often operate through complex cross-border supply chains and illicit economic systems. ^35– 37^ Global fisheries subsidies and market-driven expansion of distant-water fleets contribute to structural pressures that extend beyond Indonesia’s jurisdiction. ^33^ Thus, the first research question is addressed by demonstrating that IUU fishing in the North Natuna Sea constitutes a multidimensional threat shaped by geopolitical rivalry, global economic incentives, and transnational criminal networks.

### 3.2. Operational effectiveness of the one roof enforcement system

**Figure 2.**
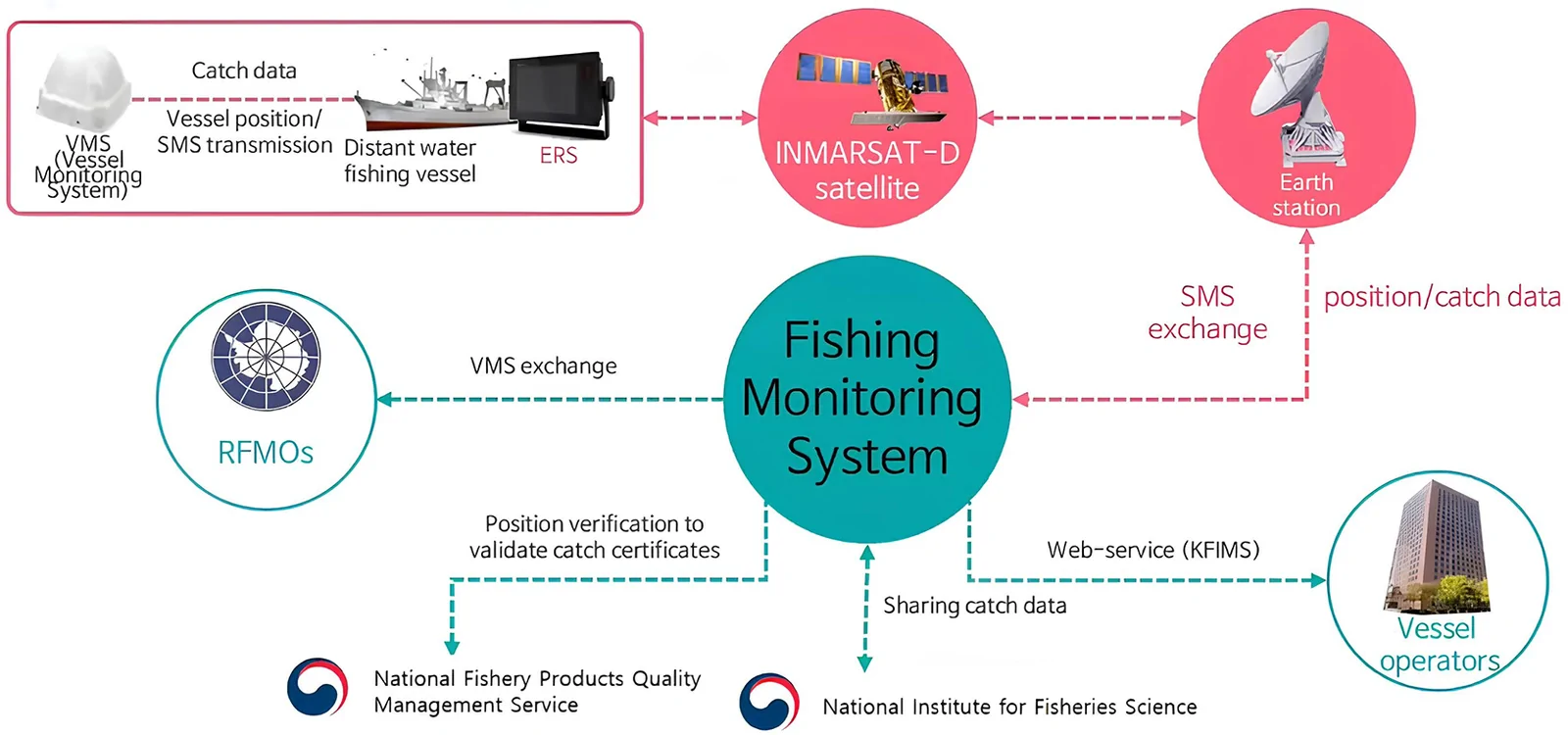
Integrated fishing monitoring and data exchange system.

Document analysis confirms that Indonesia’s legal framework for fisheries enforcement is structurally comprehensive. *Undang-Undang Nomor 45 Tahun 2009 tentang Perikanan* ^3^ provides the primary legal basis for fisheries management and sanctions. *Peraturan Pemerintah Nomor 60 Tahun 2007* regulates monitoring and surveillance mechanisms. Institutional coordination is strengthened through *Peraturan Presiden Nomor 178 Tahun 2014*, which establishes *Badan Keamanan Laut*, and *Peraturan Presiden Nomor 115 Tahun 2015*, which formalizes *Satgas 115* as a multi-agency task force. Operational procedures were further institutionalized under *Peraturan Menteri Kelautan dan Perikanan Nomor 24/PERMEN-KP/2020.* Indonesia’s fisheries monitoring architecture relies on an integrated VMS-based surveillance system linking satellite transmission, catch reporting, and inter-agency data exchange. Figure 2 illustrates the operational structure of this monitoring system.

Figure 2 illustrates the operational architecture of Indonesia’s fisheries monitoring system, including VMS transmission, satellite relay (INMARSAT-D), catch data reporting (ERS), and inter-agency data exchange with RFMO and national authorities. The system forms the technological backbone of maritime enforcement and surveillance under the multi-agency framework.

Interview findings suggest that the one roof enforcement model significantly improved inter-agency coordination, intelligence-sharing, and rapid response capacity. Informants reported clearer command structures and reduced institutional overlap compared to previous sectoral approaches. This corresponds with international evidence demonstrating that integrated monitoring, control, and surveillance (MCS) systems enhance enforcement effectiveness. ^38, 39^

**Figure 3.**
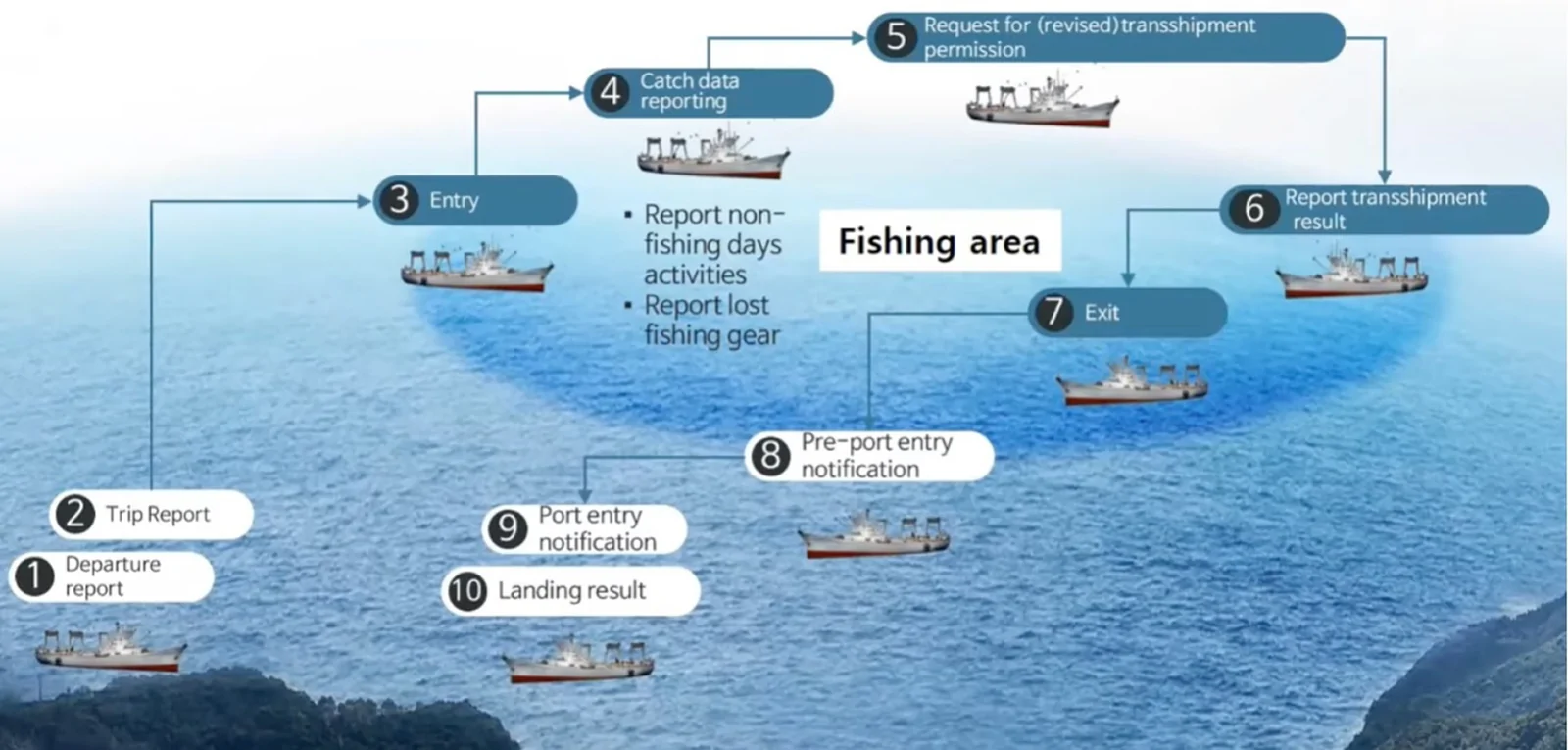
Regulatory reporting and transshipment control flow in indonesia’s fisheries monitoring system.

However, the data also reveal operational constraints. Enforcement capacity remains highly dependent on resource allocation, patrol funding, and technological integration. Compared to emerging international practices involving drones, satellite-based monitoring, and integrated ocean observing systems, ^40, 41^ technological utilization in the North Natuna Sea remains partially fragmented. Interviews indicate that while regulatory norms are robust, implementation gaps persist a pattern similarly observed in comparative studies of IUU enforcement regimes. ^42^ Beyond satellite-based detection and vessel monitoring, Indonesia’s enforcement framework relies on a structured reporting and compliance chain that regulates fishing operations from departure to landing. Figure 3 illustrates this regulatory control cycle.

While the reporting sequence provides a comprehensive compliance structure, interview findings indicate that enforcement gaps often occur at the stages of transshipment verification and port-based inspection.

### 3.3. Enforcement success versus maritime resilience

A key empirical finding concerns the distinction between short-term enforcement outputs and long-term maritime resilience. Interviewees acknowledged that intensified patrols and vessel apprehensions led to temporary reductions in illegal fishing activity. However, they also emphasized that such reductions were often cyclical and reactive. This observation is consistent with literature arguing that fisheries crime undermines broader ocean resilience and sustainable blue economy objectives. ^43^ Without structural reforms addressing local vulnerability, economic incentives, and governance participation, enforcement alone cannot generate systemic resilience. Several informants highlighted the limited integration of community-based management and co-management mechanisms in Natuna. Comparative research demonstrates that collaborative fisheries governance can enhance compliance legitimacy and ecological sustainability. ^44, 45^ Community-driven insights and participatory governance approaches have proven critical in other fisheries contexts. ^46, 47^ Sustainable maritime resilience requires diversification of economic alternatives, strengthening fisheries value chains, ^48^ and climate adaptation strategies for small-scale fishers. ^49^ In the absence of these complementary measures, enforcement risks remaining enforcement-centric rather than resilience-oriented.

### 3.4. Institutional adaptation and governance transformation

The final theme concerns institutional adaptability. While *Satgas 115* functions effectively as a coordinated enforcement mechanism, evidence suggests that it operates primarily in a reactive security framework rather than as part of a transformative governance model. Institutional effectiveness research indicates that policy durability depends on administrative capacity, inter-agency coherence, and regulatory consistency. ^50^ Lessons from quota-based and total allowable catch (TAC) pilot reforms in other jurisdictions illustrate how incremental institutional experimentation can strengthen fisheries governance sustainability. ^51^ Digitalization and integrated data systems represent additional pathways toward governance transformation. Operationalizing digital sustainability (“digitainability”) can enhance monitoring transparency and reduce enforcement costs. ^52^ However, interview data suggest that Indonesia’s enforcement system has yet to fully integrate such systemic innovations. Thus, the transition from enforcement to resilience remains incomplete. The current model improves tactical coordination but requires deeper institutional reform to achieve structural and adaptive maritime governance.

### 3.5. Alignment with research objectives

In response to the first research question, the findings confirm that IUU fishing in the North Natuna Sea is a multidimensional phenomenon shaped by geopolitical rivalry, global economic pressures, and transnational criminal structures. Regarding the second question, the multi-agency approach under *Satgas 115* demonstrates improved coordination and operational efficiency but remains resource-dependent and institutionally fragile. Concerning the third question, the policy’s contribution to maritime resilience is partial. Without integration of co-management, economic restructuring, and institutional innovation, the system risks remaining enforcement-centric rather than resilience-based.

## 4. Discussion

### 4.1. Reframing IUU fishing: from law enforcement problem to governance complexity

The findings demonstrate that IUU fishing in the North Natuna Sea cannot be adequately addressed through a purely enforcement-centric lens. While Indonesia’s multi-agency approach under *Satgas 115* enhances operational deterrence, the empirical evidence confirms that IUU fishing in this region is embedded within geopolitical rivalry, global fisheries markets, and transnational crime networks.

This aligns with scholarship emphasizing that fisheries crime increasingly operates within organized and transnational structures. ^35, 36^ The North Natuna Sea, situated within the contested South China Sea geopolitical arena, further complicates enforcement efforts. ^25, 34^ Thus, IUU fishing here represents a governance complexity problem rather than a discrete compliance violation.

From a policy evaluation perspective, the OECD framework helps clarify this complexity. The findings show that while Indonesia’s response is relevant and partially effective, it addresses primarily the security dimension of the problem. Structural drivers including global subsidy regimes, ^33^ distant-water fleet expansion, and regional governance asymmetries remain beyond the reach of national enforcement alone. Therefore, the central challenge is not the absence of enforcement capacity, but the need to transition from deterrence-based governance to systemic maritime resilience. ^53^

### 4.2. Multi-agency governance: operational coherence without structural institutionalization

The results indicate measurable improvements in horizontal institutional coherence. Asset integration, intelligence-sharing, and joint patrol mechanisms have reduced fragmentation previously observed in Indonesia’s maritime enforcement architecture.

**Figure 4.**
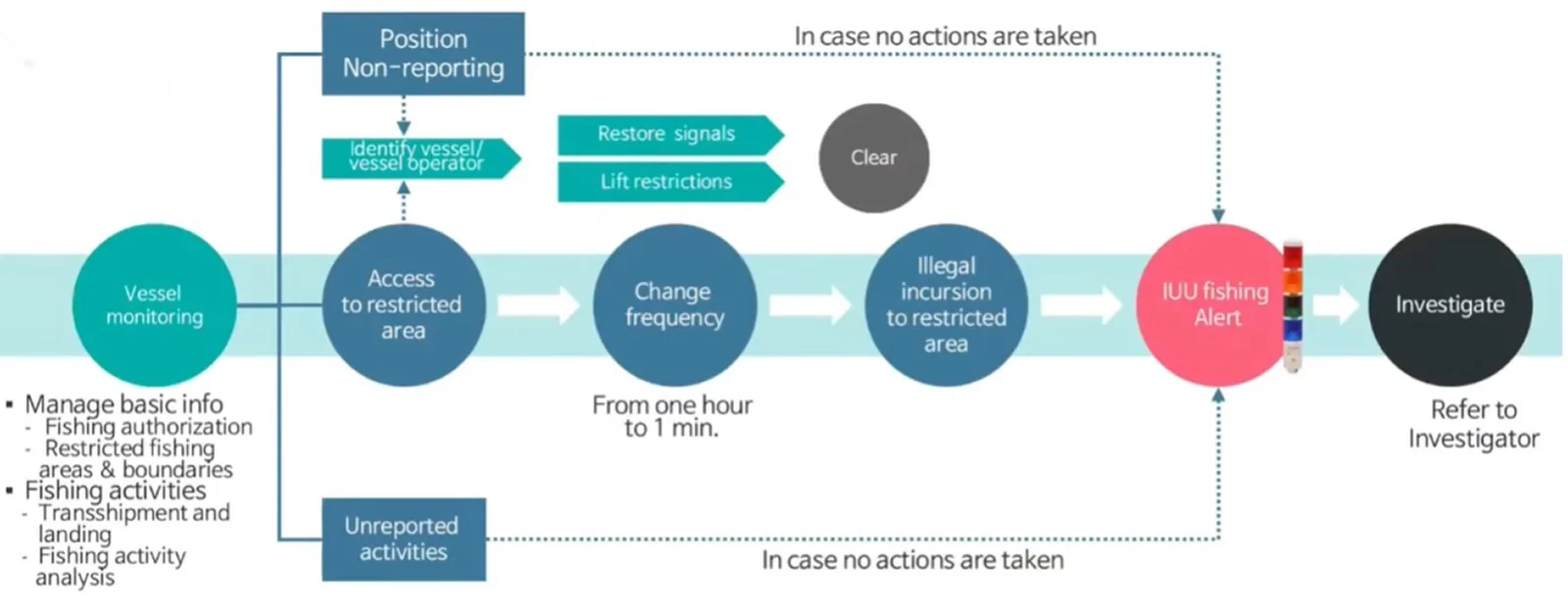
Enforcement escalation logic and absorptive capacity in maritime governance.

This confirms international evidence that integrated monitoring, control, and surveillance (MCS) systems enhance enforcement effectiveness. ^38, 39^ The one roof enforcement model represents a significant institutional innovation within Indonesia’s maritime governance system. While Indonesia’s monitoring and reporting architecture is structurally comprehensive, the enforcement response largely follows a reactive escalation logic. Figure 4 illustrates the monitoring-to-investigation escalation pathway embedded within the current enforcement system.

The system demonstrates strong absorptive capacity through rapid alert generation and investigative referral. However, it lacks embedded feedback loops that would institutionalize adaptive learning and preventive governance mechanisms.

However, the empirical findings reveal that coordination remains dependent on centralized political prioritization and extraordinary policy measures. Such dependency reflects what can be described as operational coherence without structural institutionalization. Comparative research suggests that durable governance systems require embedded institutional mechanisms rather than ad hoc coordination frameworks. ^42^ Without stable financing structures, long-term integration of technological systems, and vertical policy alignment with coastal governance actors, coordination risks regression when political momentum declines. Thus, the Indonesian case highlights a broader governance paradox: multi-agency consolidation can improve short-term coherence but does not automatically produce institutional resilience.

### 4.3. Effectiveness and the limits of deterrence

The study confirms that intensified patrols and vessel apprehensions reduce illegal fishing activity during peak enforcement periods. However, the cyclical re-emergence of IUU activity when patrol intensity declines suggests that deterrence effects remain input-dependent. This pattern reinforces findings from global fisheries enforcement literature that surveillance intensity alone cannot produce structural compliance transformation. ^38^ Deterrence may suppress illegal behavior temporarily but does not eliminate underlying economic incentives. Moreover, technological modernization such as drone surveillance, satellite monitoring, and integrated observing systems remains unevenly institutionalized in Indonesia. ^41^ In contrast, digital governance integration is increasingly recognized as central to sustainable maritime monitoring systems. ^52^ Therefore, effectiveness in the North Natuna Sea should be understood as operationally significant but systemically fragile.

### 4.4. Maritime resilience: absorptive strength, adaptive weakness

Applying a maritime resilience framework provides deeper insight into policy limitations. The findings indicate strong absorptive capacity: Indonesia can respond rapidly to maritime violations through coordinated operations. This reflects significant state capability in mobilizing enforcement resources under crisis conditions.

However, adaptive capacity remains limited. There is insufficient institutionalized learning, limited integration of fisheries co-management mechanisms, and weak engagement with coastal communities. Comparative research demonstrates that participatory governance strengthens long-term compliance and ecological sustainability. ^44, 45^ Transformative capacity shifting from enforcement-driven control to integrated fisheries governance also remains underdeveloped. Successful governance reforms elsewhere, such as quota-based experimentation and total allowable catch pilots, ^51^ illustrate how incremental institutional transformation can reduce enforcement dependency. In the North Natuna Sea, enforcement remains the dominant policy instrument, while structural fisheries management reform, economic diversification strategies, and regional diplomatic engagement remain secondary. Thus, Indonesia’s maritime governance system appears to be in a transitional phase between deterrence and resilience.

### 4.5. From enforcement to resilience

Ultimately, the Indonesian experience in the North Natuna Sea illustrates both the strengths and limitations of enforcement-centric policy. The multi-agency consolidation under *Satgas 115* constitutes a necessary foundation for maritime security in a contested region. Yet enforcement alone cannot generate durable maritime resilience. Building long-term resilience requires institutional stability, governance innovation, socio-economic integration, and regional cooperation. The transition from enforcement to resilience is therefore not merely operational but structural. This transition represents the central conceptual contribution of this article: demonstrating that combating IUU fishing in contested maritime spaces demands not only stronger patrols, but smarter governance systems capable of learning, adapting, and transforming over time.

Table 1 presents enforcement outcomes in the North Natuna Sea from 2020 to May 2025, showing 147 apprehended vessels (85 domestic and 62 foreign) and an estimated IDR 2.1 trillion in prevented state losses. The data indicate strong short-term deterrence capacity under Indonesia’s integrated maritime enforcement operations, particularly during joint patrols coordinated through *Satgas 115.* The significant share of foreign vessels confirms the transnational and security-sensitive nature of IUU fishing in the North Natuna Sea. However, fluctuations in capture numbers suggest that enforcement performance remains closely tied to operational intensity and resource allocation.

**Table 1.** Enforcement outcomes and institutional implications in the north natuna sea (2020–May 2025).

Year/Period	Number of captured vessels	Type (domestic/foreign)	Estimated state losses prevented (IDR)	Main contributing operations	OECD dimension most evident	Resilience capacity reflected
2020–2024 (Jan–May)	115	70 domestic/45 foreign	1.3 trillion	PAM LNU; Joint Operations (KKP, TNI AL, Bakamla) under *Satgas 115*	Effectiveness; Relevance	Strong Absorptive Capacity (deterrence & rapid response)
2025	32	30 domestic/2 foreign (Vietnam)	774.3 billion	Bakamla-led maritime patrol operations	Effectiveness; Coherence (horizontal)	Moderate Absorptive Capacity; Limited Adaptive Mechanism
**Total (2020–May 2025)**	**147**	**85 domestic/62 foreign**	**2.1 trillion**	Integrated Maritime Security Operations (multi-agency model)	Relevance; Effectiveness; Partial Impact	Absorptive > Adaptive > Transformative

**Source**: ^54, 55^

## 5. Conclusion

This study set out to evaluate Indonesia’s one roof enforcement system in addressing IUU fishing in the North Natuna Sea and to assess its contribution to long-term maritime resilience. Using a qualitative case study approach and the OECD policy evaluation framework, the research demonstrates that while the multi-agency model under *Satgas 115* represents a significant institutional innovation, its impact remains predominantly enforcement-centric and operational rather than structurally transformative.

Empirically, the findings confirm that IUU fishing in the North Natuna Sea constitutes a multidimensional governance challenge shaped by geopolitical contestation, transnational economic pressures, institutional fragmentation, and ecological vulnerability. The multi-agency consolidation model improves horizontal coordination and enhances short-term deterrence capacity. In periods of intensified joint operations, foreign fishing vessel presence declines, and state visibility in contested waters increases. However, the study also reveals that policy effectiveness remains input-dependent and fluctuates with patrol intensity, fiscal allocation, and political prioritization. Efficiency constraints, technological gaps, and limited vertical integration with local governance structures further constrain policy durability. While Indonesia demonstrates strong absorptive capacity in responding to maritime violations, adaptive and transformative capacities remain underdeveloped. From an OECD evaluation perspective, the policy scores relatively high on relevance and short-term effectiveness but exhibits limitations in efficiency, long-term impact, and sustainability. The findings therefore indicate a structural gap between enforcement outputs and systemic maritime resilience.

The central contribution of this article lies in reframing IUU fishing in contested maritime spaces as a governance complexity problem rather than merely a compliance issue. By integrating policy evaluation criteria with a maritime resilience framework, this study advances understanding of how enforcement-driven approaches can serve as necessary foundations but insufficient endpoints in fisheries governance reform. For Indonesia and other large archipelagic states facing similar challenges, the transition from enforcement to resilience requires institutionalized inter-agency coordination, technological modernization, integration of fisheries management reform, engagement with coastal communities, and strengthened regional maritime diplomacy. Without such structural adaptation, enforcement mechanisms risk remaining reactive rather than transformative. Ultimately, combating IUU fishing in the North Natuna Sea is not solely about increasing patrols or apprehending vessels; it is about building a governance system capable of learning, adapting, and sustaining maritime order in an increasingly complex geopolitical and socio-ecological environment.

### 5.1. Policy implications

The findings suggest four strategic policy directions:

a. Institutionalization of coordination mechanisms beyond extraordinary measures, including permanent inter-agency funding frameworks;
b. Integration of technological modernization into a unified maritime surveillance architecture;
c. Strengthening vertical coherence through structured involvement of regional governments and coastal communities;
d. Linking enforcement to broader fisheries governance reform and regional maritime diplomacy.

Such shifts would move Indonesia from reactive deterrence toward systemic resilience.

### 5.2. Theoretical contribution

This study contributes to maritime governance scholarship in three ways:

a. It empirically demonstrates how a one roof enforcement system functions within a geopolitically contested maritime environment.
b. It operationalizes the OECD evaluation framework within a maritime security context, revealing distinctions between output effectiveness and systemic sustainability.
c. It advances maritime resilience theory by differentiating absorptive, adaptive, and transformative capacities in fisheries governance.

By reframing IUU fishing as a governance complexity problem rather than solely a compliance issue, the study extends international debates on fisheries crime, maritime security, and sustainable ocean governance.

## Data Availability

The data supporting the findings of this study are openly available in Zenodo. The dataset is associated with the article *“From Enforcement to Resilience: Evaluating Indonesia’s One Roof Enforcement System to Combat Illegal Fishing in the North Natuna Sea”* and can be accessed via the following DOI: https://doi.org/10.5281/zenodo.19662884. ^56^ All data are distributed under the **Creative Commons Attribution 4.0 International (CC BY 4.0)** license. Data in the repositoty include:
1. Data Table 2. Interview Guide
