# Supplementary material for: From Enforcement to Resilience: Evaluating Indonesia's One Roof Enforcement System to Combat Illegal Fishing in the North Natuna Sea
Source: F1000Res. 2026 May 12;15:709. [Version 1] doi: 10.12688/f1000research.179511.1 (PMC13560702; doi:10.12688/f1000research.179511.1)
Supplement: Peer review history

# Reviewer report: Reviewer response for version 1

**DOI:** 10.5256/f1000research.198032.r499902

**Published:** 2026-08-12

**Authors:** Anup Phayal

**Affiliations:** 1 University of North Carolina at Wilmington, Wilmington, North Carolina, USA

I appreciate the opportunity to review the paper entitled "From Enforcement to Resilience: Evaluating Indonesia’s One Roof Enforcement System to Combat Illegal Fishing in the North Natuna Sea" in *F1000 Research*. The paper addresses the important issue of illegal fishing in Indonesia and examines the effects of the recently adopted unified enforcement approach. I hope the authors find my comments below helpful as they revise the manuscript.

  

1. The paper highlights the unified approach to tackling illegal fishing through the establishment of Task Force 115. It employs semi-structured interviews with key informants from maritime enforcement institutions, along with document analysis. The findings show that the unified approach is effective in addressing the multidimensional nature of illegal fishing in the North Natuna Sea, while also raising questions about its long-term viability. Below, I have some constructive comments and questions.
2. In my view, the authors could elaborate slightly more on the unified approach itself and explain how it differs from the previous sectoral approach. For instance, they discuss its legal evolution in paragraph 3, and the subsequent paragraph briefly mentions achievements such as “high-profile vessel seizures and scuttling operations, and a temporary reduction in the presence of foreign fishing vessels.” However, as a reader, I was curious to understand the structural consequences after its adoption: what exactly changed. Did its adoption mean changes in command or organizational structure? Were there any changes in the decision-making process? Which institutions were added or excluded?
3. Section 3.2 on Operational effectiveness was very informative, particularly how the coordination process changed after Satgas 115. However, one of the most important aspects of this section could be further developed. The authors note that the interview findings reveal gaps in transshipment verification and post-based inspection. While the issue is mentioned, I feel that providing some additional explanation would be helpful. What are these gaps? What, where, and how exactly do they occur in practice? A brief discussion or example(s) would strengthen the section.
4. Similarly, the authors have an opportunity to further highlight an important issue in Section 3.4, "Institutional adaptation and governance transformation." They draw on institutional effectiveness research, TAC reforms, digitalization, and integrated data systems to show what has worked better elsewhere. However, this section ends somewhat abruptly, suggesting that the interview data shows “Indonesia’s enforcement system has yet to fully integrate such systemic innovations.” This finding would be much more meaningful if they expanded on it slightly: what systems currently exist in Indonesia, and which specific elements or capabilities are still lacking.
5. Please briefly explain Figure 4 in the main text so the readers can interpret the key message.
6. Lastly, the authors repeatedly emphasize a systemic approach to addressing illegal fishing, noting that enforcement alone may just be a short-term solution rather than a sustainable one. Recent research indicates that relying solely on enforcement measures can lead to the displacement effect, increasing other criminal activities, such as piracy. Including these recent works into the literature review could further enhance the paper.
7. Overall, it was a pleasure to read this research and gain insight into Indonesia’s policy approach to addressing illegal fishing in the North Natuna Sea. The paper makes an excellent contribution to the literature, and I hope these comments are helpful as the authors refine their work.

Is the work clearly and accurately presented and does it cite the current literature?

Yes

If applicable, is the statistical analysis and its interpretation appropriate?

Not applicable

Are all the source data underlying the results available to ensure full reproducibility?

No source data required

Is the study design appropriate and is the work technically sound?

Yes

Are the conclusions drawn adequately supported by the results?

Yes

Are sufficient details of methods and analysis provided to allow replication by others?

Yes

Reviewer Expertise:

Maritime Security, International Relations

I confirm that I have read this submission and believe that I have an appropriate level of expertise to confirm that it is of an acceptable scientific standard.

# Reviewer report: Reviewer response for version 1

**DOI:** 10.5256/f1000research.198032.r505421

**Published:** 2026-08-03

**Authors:** Rendi Prayuda

**Affiliations:** 1 Universitas Islam Riau, Pekanbaru, Indonesia

This manuscript addresses an important issue concerning the governance of Illegal, Unreported, and Unregulated (IUU) fishing in the North Natuna Sea through Indonesia's multi-agency enforcement system. The abstract is well organized, logically structured, and clearly communicates the research objective, methodology, principal findings, and policy implications. The topic is timely and relevant for scholars of maritime governance and public policy. Overall, the manuscript is suitable for indexing after minor revisions.

 The background effectively introduces the governance challenge but could articulate the research gap more explicitly.

 Current statement:

 "...the extent to which this approach contributes to long-term maritime resilience remains underexplored."

 Suggested improvement:

 Briefly indicate **what previous studies have predominantly emphasized** (e.g., maritime security, law enforcement effectiveness, sovereignty protection) and explain how this study differs by integrating the OECD policy evaluation framework with the maritime resilience perspective.

 This will enhance the originality of the research contribution.

 The manuscript presents a meaningful contribution to the literature on maritime governance and public policy. The analytical framework combining the OECD Policy Evaluation Framework with Maritime Resilience offers a valuable perspective for evaluating multi-agency enforcement systems. The suggested revisions are intended to improve clarity, methodological justification, and the articulation of the manuscript's scholarly contribution without requiring substantial additional analysis.

Is the work clearly and accurately presented and does it cite the current literature?

Yes

If applicable, is the statistical analysis and its interpretation appropriate?

Yes

Are all the source data underlying the results available to ensure full reproducibility?

Yes

Is the study design appropriate and is the work technically sound?

Yes

Are the conclusions drawn adequately supported by the results?

Yes

Are sufficient details of methods and analysis provided to allow replication by others?

Yes

Reviewer Expertise:

International Security, Transnational Organized Crime, Border Security

I confirm that I have read this submission and believe that I have an appropriate level of expertise to confirm that it is of an acceptable scientific standard.

# Reviewer report: Reviewer response for version 1

**DOI:** 10.5256/f1000research.198032.r505419

**Published:** 2026-07-28

**Authors:** Mohd Hazmi Mohd Rusli

**Affiliations:** 1 Universiti Sains Islam Malaysia, Nilai, Malaysia

Overall, I enjoyed reading this manuscript. It addresses an important and timely issue, and I believe it makes a valuable contribution to the literature on maritime governance and IUU fishing. One of the main strengths of the paper is that it moves beyond the traditional discussion of IUU fishing as simply a law enforcement issue. Instead, it examines the issue from a broader governance perspective by combining the OECD policy evaluation framework with the concept of maritime resilience. This gives the paper a fresh perspective and adds to its originality.

 The research is well designed and the methodology is appropriate for the research questions. The use of interviews, document analysis, and observation provides sufficient evidence to support the findings. The paper is also well organised, making it easy for readers to follow the discussion from the research problem, methodology, findings, and finally to the policy implications.

 I also appreciate that the authors do not simply measure the success of Satgas 115 by the number of vessels apprehended. Instead, they distinguish between short-term enforcement success and long-term maritime resilience. This provides a more balanced assessment of Indonesia's one roof enforcement system and highlights that effective enforcement alone is not enough to achieve sustainable maritime governance. I believe this is one of the strongest contributions of the paper.

 The discussion is thoughtful and goes beyond simply explaining the findings. The authors successfully relate their results to broader issues of maritime governance, institutional coordination, and regional security. The conclusions are also practical and provide useful policy recommendations that may benefit not only Indonesia but also other coastal and archipelagic states facing similar challenges.

 I have only a few suggestions which the authors may wish to consider: 

 First, the paper could discuss the wider South China Sea situation in a little more detail. Since the North Natuna Sea is closely linked to regional geopolitical developments, a stronger discussion of this aspect would further strengthen the international significance of the paper.

 Second, it may be useful to include a brief comparison with similar multi-agency enforcement systems in other countries. Even a short discussion would help readers better understand the strengths and uniqueness of Indonesia's approach.

 Third, the authors may consider providing slightly more information about the interview participants, such as their institutional background or level of experience, while maintaining confidentiality. This would improve the transparency of the research methodology.

 Finally, the policy recommendations could be made a little more specific by explaining how some of the proposed reforms, particularly those relating to technology, community participation, and institutional coordination, could be implemented in practice.

 The comments above are offered only as constructive suggestions for the authors to consider in further improving an already strong manuscript. They should not be taken as an indication that the paper is unsuitable for publication. In my view, this is a well-written, well-researched, and relevant study that makes a meaningful contribution to the field. I therefore support its publication after the authors have considered the above suggestions.

Is the work clearly and accurately presented and does it cite the current literature?

Yes

If applicable, is the statistical analysis and its interpretation appropriate?

Yes

Are all the source data underlying the results available to ensure full reproducibility?

Yes

Is the study design appropriate and is the work technically sound?

Yes

Are the conclusions drawn adequately supported by the results?

Yes

Are sufficient details of methods and analysis provided to allow replication by others?

Yes

Reviewer Expertise:

Law of the Sea, Maritime Policy, Marine Environmental Law, Public International Law

I confirm that I have read this submission and believe that I have an appropriate level of expertise to confirm that it is of an acceptable scientific standard.
